# Aortic Rupture During Surgical Management of Tubercular Spondylodiscitis

**DOI:** 10.7759/cureus.2255

**Published:** 2018-03-01

**Authors:** Phani Krishna Karthik Yelamarthy, Rajat Mahajan, Tarush Rustagi, Vikas Tandon, Gururaj Sangondimath, Harvinder Singh Chhabra

**Affiliations:** 1 Spine Surgery, Indian Spinal Injuries Center, New Delhi; 2 Department of Spine Surgery, Indian Spinal Injuries Center, New Delhi

**Keywords:** aortic rupture, complication, spine surgery, tubercular spondylodiscitis, pvcr

## Abstract

Aortic rupture is a rare but possible complication during spine surgery. It may manifest as severe intraoperative hemorrhage or present in a delayed manner after the formation of an aneurysm or an arteriovenous fistula. Though it is commonly encountered during anterior surgeries involving the surgical field close to the thoracic or abdominal aorta, it can also occur during a posterior surgery. Aortic injury could be associated with surgeries ranging from the commonly performed pedicle screw instrumentation to a complex three-column osteotomy. It can also occur, as in the reported case, while performing complex procedures in the presence of a pre-existing aneurysm or aortic adhesions due to coexisting infectious or inflammatory pathologies. The treatment options for such aortic ruptures range from open repair to endovascular stenting techniques. We discuss a case of an aortic rupture that occurred during a posterior vertebral column resection (PVCR) procedure performed on a 58-year-old female with spastic paraparesis secondary to tuberculous spondylodiscitis and the lessons learnt.

## Introduction

Aortic rupture during a spine surgery is a known yet disastrous complication. It can be fatal and lead to the death of a patient on the operative table. It can occur due to pre-existing aortic aneurysms or aortic adhesions due to coexisting infectious or inflammatory pathologies, during anterior surgery while working around big vessels and rarely during posterior spinal surgery. The incidence depends on the anatomical region, surgical approach, primary pathology, implant-related issues, and the surgical expertise [1]. Injury to the aorta usually presents as immediate intraoperative hemorrhage, or it can present as a late manifestation due to the formation of an aneurysm or arteriovenous fistula [2]. We describe here a case of aortic injury during a posterior vertebral column resection (PVCR) procedure for tubercular thoracolumbar kyphotic deformity.

## Case presentation

History

**Figure 1:**
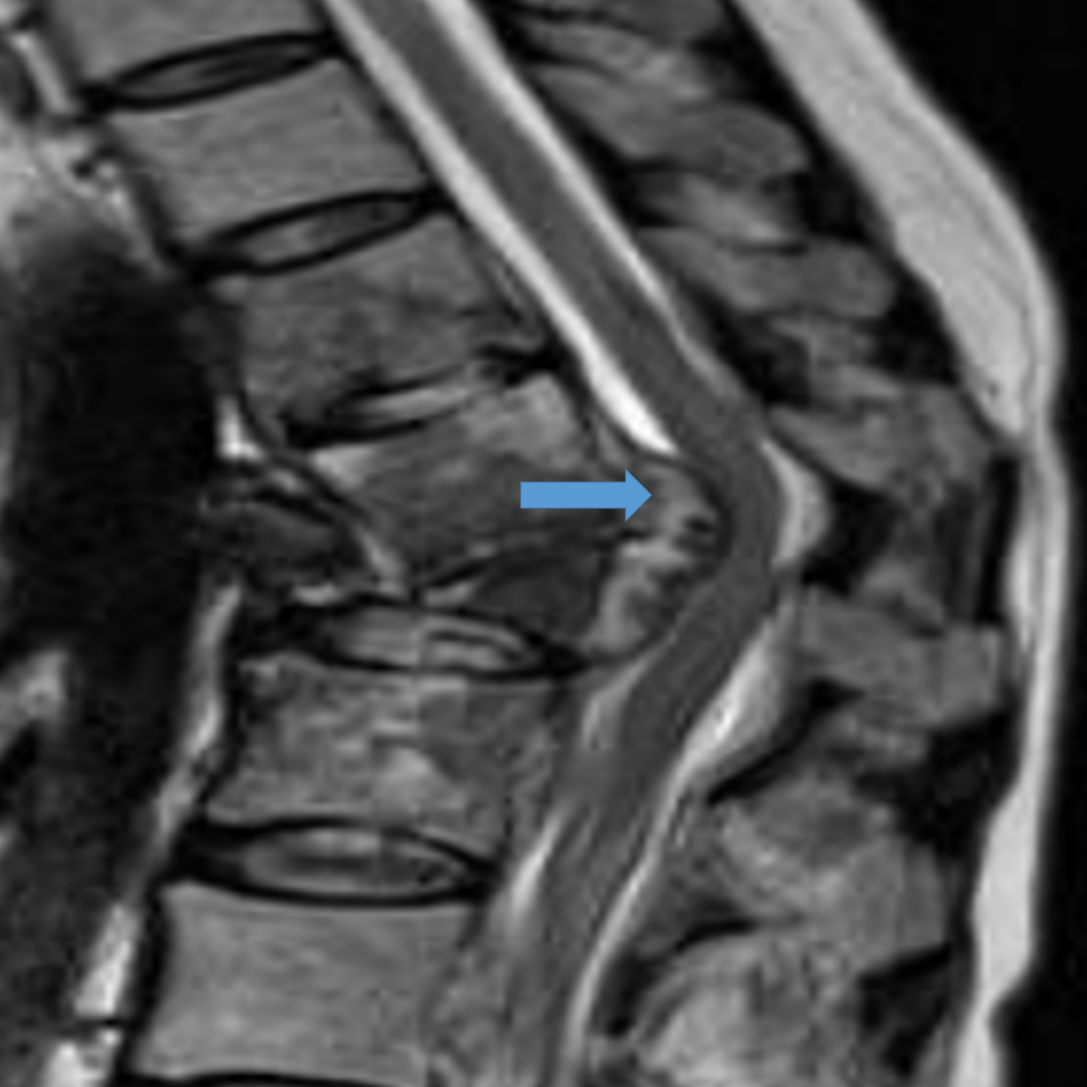
T2 MR sagittal image showing angular kyphosis at D12-L1 with epidural soft tissue component (block arrow) and accompanying cord compression. MR - magnetic resonance

A 58-year-old female presented with a gradually progressive, painful kyphotic deformity of the mid back of five months duration associated with progressive spastic paraparesis, with onset two weeks before presenting to our clinic. On examination there was a tender gibbus deformity in the thoracolumbar junction with spastic paraparesis and exaggerated deep tendon reflexes. Her hematological parameters showed leukocytosis (14,000 cells/cubic millimeter) with an elevated erythrocytic sedimentation rate (70 mm/hr). X-rays showed a focal kyphotic deformity at the thoracolumbar junction. Magnetic resonance imaging (MRI) was suggestive of spondylodiscitis at T12-L1 with epidural soft tissue components (Figure 1), likely secondary to a tubercular infective pathology.

Surgery and postoperative course

Surgical planning was done to perform a PVCR of T12 and L1. After the surgical exposure, pedicle screws were inserted followed by a wide laminectomy of T11-L2, and a temporary rod was placed on the right side. This was followed by removal of the facets and pedicles of T12 and L1. At this time, vertebral body resection was started. During removal of the anterior part of the body, torrential arterial bleeding was encountered while removing one of the sequestered vertebral bone fragments. The anesthesia team was alerted of a probable aortic injury. The wound was packed, the rod was placed on the other side, rapid closure was done, and the vascular team was called in. Hemodynamics was maintained with rapid infusion of blood products and ionotropic support. In the right lateral position, a thoracoabdominal anterior approach was performed and the site of injury was identified. The aorta was found adhered to the surrounding structures. The source of bleeding was traced to the aorta where a 4 * 4 mm circumscribed irregular defect with friable inverted margins was identified. The defect was far posterolateral and was not freely accessible. After a couple of failed attempts due to difficult accessibility and friable margins, the vascular surgeon was able to pass a couple of sutures sealing the defect. There was no further active bleeding. Surgical procedure was then abandoned as the expected blood loss was around 4000 cc. The plan was for the vascular surgeon to do a semi-elective stenting procedure the next day to reinforce the repair. The patient was shifted to the intensive care unit but she expired the next day.

## Discussion

Injury to thoracic or abdominal aorta has been reported to occur following spinal surgical procedures because of surgical armamentarium or pathologies distorting the normal anatomy of the aorta. Aortic injury has been reported during surgical exposure, insertion of pedicle screws, vertebroplasty, lumbar discectomy, lumbar interbody fusions, and during three-column osteotomies [3-6].

Spinal pathologies such as infections and inflammatory diseases result in adhesions of the aorta to its vicinity thus reducing the mobility and making them more prone to rupture spontaneously or iatrogenically or after minor trauma, especially if there is a pre-existing aneurysm [7]. Malposition of pedicle screws, even though under-reported, can be associated with aortic injury [5].

**Figure 2:**
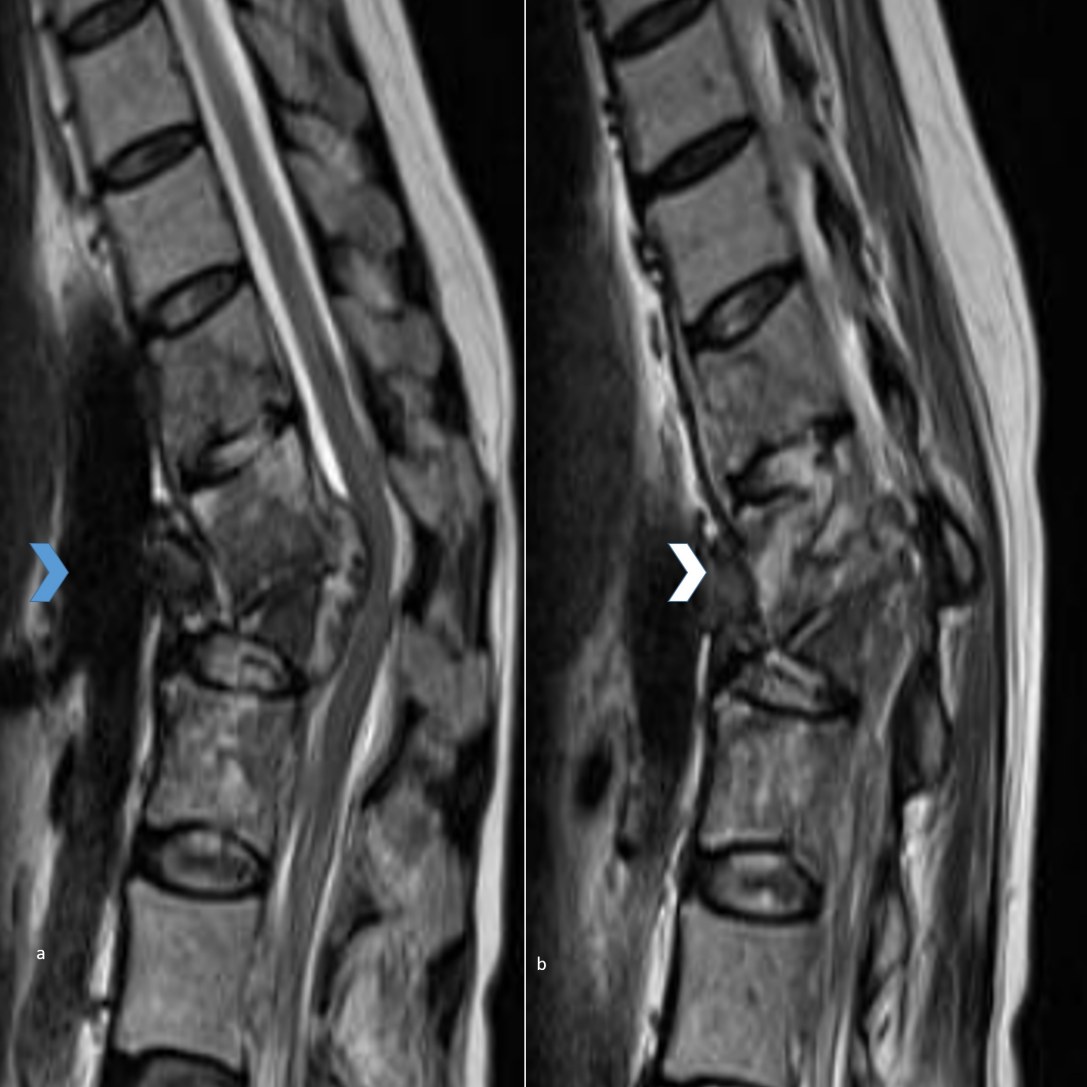
Figures 2a and 2b showing midsagittal and parasagittal T2 MR sequences showing a sequestered fragment adherent to the aorta (aorta and the sequestered fragment are marked by blue and white bold arrows, respectively). MR - magnetic resonance

Our case was a post-infectious kyphotic deformity with spastic paraparesis of five month duration. The treatment of infectious spinal deformity has undergone a radical change during the last two decades. Earlier surgeons performed an anterior approach for most cases of spondylodiscitis associated with neurological deficit and implants were seldom used [8]. With the advent of pedicle screws, the posterior approach has gained popularity allowing deformity correction and anterior column reconstruction [9]. In order to address the deformity and perform adequate decompression, we opted for a single stage PVCR. On retrospective review of the images, we identified that one of the bone fragments was possibly stuck to the aorta (Figures 2a-2b).

During corpectomy, when this fragment was being fished out, there was a rupture of the aortic wall. Though we managed to repair the injured area of aorta, the patient succumbed on the next day due to complications of blood loss and disseminated intravascular coagulation (DIC) due to massive blood transfusion. 

Spinal infections have been reported to be associated with spontaneous aortic rupture with or without associated aneurysmal formation [7]. Cage migration has been most commonly associated with vascular injury following three-column osteotomies [10, 6]. However, there are no previous reports of aortic injury during a three-column osteotomy for spondylodiscitis. We report a case of aortic injury following adhesions secondary to infectious spondylodiscitis, which turned to be of tubercular etiology (Tb PCR positive). MRI helps to identify aneurysmal formation, proximity of great vessels to the vertebral body, and to assess if any bony or soft tissue fragment is abutting the great vessels. Our case had no associated aneurysm formation, but in retrospect, we identified a bony fragment adhered to the aorta. Our case highlights the importance of critically evaluating the MRI, being cognizant of this catastrophic complication associated with tubercular spondylodiscitis. We feel that careful preoperative observation and further evaluation with arteriography may have made us more careful in performing corpectomy by leaving the possibly adhered fragment in situ.

## Conclusions

Aortic rupture while performing a PVCR for spondylodiscitis has not been described before. Doing PVCR in spondylodiscitis can be a problem since long standing infection can be associated with the adherence of great vessels to the vertebral body. Our case highlights the significance of carefully evaluating the surrounding structures on the MRI preoperatively to look for adherent fragments to the aorta and leaving such fragments in situ during surgical resection to prevent surgical catastrophes.
